# Intraabdominal and retroperitoneal soft-tissue sarcomas - outcome of surgical treatment in primary and recurrent tumors

**DOI:** 10.1186/1477-7819-8-81

**Published:** 2010-09-12

**Authors:** Ane S Sogaard, Jacob M Laurberg, Mette Sorensen, Ole S Sogaard, Pal Wara, Peter Rasmussen, Soren Laurberg

**Affiliations:** 1 Department of Surgery P, Aarhus University Hospital, Aarhus, Denmark; 2 Department of infectious Diseases, Aarhus University Hospital, Aarhus, Denmark; 3 Sarcoma Center, Aarhus University Hospital, Aarhus, Denmark

## Abstract

**Background:**

Surgery is the only curative treatment for intraabdominal and retroperitoneal sarcoma (IaRS). Little is known about how to treat patients with recurrence. We here report the outcome in primary and recurrent sarcoma treated at the Sarcoma Center in Aarhus, Denmark.

**Methods:**

All patients evaluated for IaRS from June 1998 to May 2008 were enrolled and data on symptoms, signs, means of diagnosis, extent of surgery, perioperative complications, mortality and long time survival were registered. Primary and first-recurrence sarcomas were analyzed separately.

**Results:**

Sixty-five of 73 primary and 22 of 28 first-recurrence IaRS had surgery. Fifty-three (82%) and 11 (50%) patients achieved radical R0 resection. Age and radicality of surgery were independent predictors of death, while recurrence of sarcoma was not. Perioperative mortality was 2.3%. 5-year survival was 70.2% for primary and 51.8% for first-recurrent sarcomas. However, patients with radical surgery had 5-year survival of over 70% in both the primary and recurrent group.

**Conclusions:**

The radicality of surgery is the most important prognostic factor. Patients with recurrence have an equally good prognosis as those with primary sarcoma if radicality is achieved and such surgery should not be considered only as a palliative effort.

## Background

Soft tissue sarcomas are a heterogeneous group of malignant tumors originating from mesenchymal cells. They constitute just under 1% of all cancers [1], corresponding to only 9000 new cases annually in US, and 1500 in UK [1,2]. Approximately 20% of soft tissue sarcomas arise from intraabdominal or retroperitoneal cells [3], and the three most prevalent histopathological types are gastrointestinal stromal tumor (GIST), leiomyosarcoma, and liposarcoma [4-6]. However, any mesenchymal cell, is capable of malignant transformation, and more than 100 different histopathological types of sarcoma have been described [7,8].

Diagnosing intraabdominal and retroperitoneal sarcomas (IaRS) is often difficult since the signs and symptoms are often discreet and uncharacteristic. General symptoms are common, and depending on tumor site, haemorrhage, ascites, pressure symptoms, and pain may be present. Consequently, the diagnosis is often made at an advanced stage when the tumor has reached a considerable size.

The final diagnosis is usually made by imaging modalities such as MR-, CT-, or ultrasound scans. It is recommended, that preoperative biopsies are performed using a fine needle because of the risk of spreading through tumor seeding, also considering the puncture route [9,10]. The literature on outcome in particular in recurrent sarcomas with modern surgical treatment is scarce. The aim of the present prospective cohort study is to report the outcome of surgical treatment of primary as well as recurrent sarcoma in our center over the last 10 years.

## Patients and methods

From June 1998 to May 2008 all patients over 18 years of age with IaRS examined at the sarcoma center at the surgical department P, Aarhus University Hospital, were registered consecutively. The center is a large elective surgery department which also has extensive experience with other forms of advanced abdominal surgery procedures. It covers specialized surgical functions for western Denmark, an area with approximately 2 million inhabitants. During this period, all peripheral surgical departments in the area began referring sarcoma patients to the center for diagnosis, evaluation, and surgery.

Data on primary and recurrent tumor were collected including preoperative symptoms and diagnostic methods. Patients with primary sarcoma or any first recurrence of sarcoma were included in the statistical analysis while patients with more than one recurrence were excluded from the study. In the following, the term recurrent disease refers to patients having their first recurrent disease unless otherwise stated. Variables related to the pre-, peri- and postoperative period were collected and included: Age, gender and symptoms, preoperative diagnosis, preoperative biopsy (yes/no), metastasis (yes/no), site of origin, preoperative medical treatment, tumour (primary/1st recurrence), operability (operable/inoperable), resection of adjacent organs, radicality (R0 = macro- and microscopically radical resection, R1 = macroscopically, but not microscopically radical, and R2 = macroscopical residual tumour tissue, local or distant), histopathological diagnosis, postoperative complications, and perioperative mortality, as well as longterm survival. None of the tumors types (including GIST tumors) received neoadjuvant treatment. R1 and R2 GIST tumors received Imatinib as palliative treatment. R1 and R2 liposarcomas were also offered palliative treatment.

Since 1968, all Danish residents have been assigned a unique 10-digit personal identification number by the Central Office of Civil Registration. Patients are identified by this number during all contacts with the healthcare system. Likewise, all deaths are registered using this number. Thus, we were able to trace the exact date of death for every patient. Patients were also linked with all hospital discharge registries which collect data of hospitalizations since 1977. Patients were followed until 31 December 2008.

We registered a total of 114 contacts in 96 patients. Of the 114 contacts, 73 presented with a primary tumor while 28 had a first recurrence of tumor. 13 of the contacts had second- or more recurrencies and were excluded, so the population included in the analysis was 73 primary and 28 recurrent sarcomas for a total of 101 contacts.

Sixty-five of 73 (89%) primary sarcomas had surgery, and 22 of 28 (79%) patients with recurrent disease were considered operable (p = 0.11). Of these 87 operations, R0- resection was achieved in 51 of 65 (78%) of the patients with primary tumor and 11 of 22 (50%) of the patients with first-recurrence (p < 0.01).

Histopathologically, 39% of IaRS were GIST, liposarcomas constituted 18%, whereas relatively few leiomyosarcomas were found (11%). Thirty percent of the tumors had a different histological type than these three types. These consisted of more than 20 different rare histopathological types (Data not shown).

Median time of follow-up was 2.94 years (interquartile range: 0.97-4.65). Baseline characteristics are shown in Table 1. All operations were performed by the same 3 surgeons.

**Table 1:** Baseline characteristics, symptoms, and signs

	Primary Tumor (n = 73)	1st Recurrence (n = 28)	p
Gender			0.267
Male	39(53%)	11(39%)	
Female	34(47%)	17(61%)	
Age			
Mean years, sd	58.0 ± 15.0	55.1 ± 12.8	0.365
Median	60.0	56.0	
Median 25%	50.0	45.5	
Median 75%	68.0	61.5	
Analgetics*			0.211
None	54 (75%)	16 (59%)	
Non-opioids	16 (22%)	9 (33%)	
Opioids	2 (3%)	2 (7%)	
Enlarged Abdomen*			0.596
No	57 (79%)	20 (74%)	
Yes	15 (21%)	7 (26%)	
Bleeding*			0.126
No	50 (69%)	25 (93%)	
Intraabdominal	5 (7%)	0	
Upper GI	5 (7%)	0	
Lower GI	12 (17%)	2 (7%)	
Obstruction*			1.000
No	60 (83%)	23 (85%)	
Postprandial pain	7 (10%)	2 (7%)	
Ileus	5 (7%)	2 (7%)	
Palpable abdominal mass*			0.647
No	29 (40%)	9 (32%)	
Yes	43 (59%)	17 (61%)	
Metastases*			
No	69 (95%)	20 (71%)	0.010
Yes	3 (4%)	6 (21%)	

* Exact data not available on all patients

### Statistical analyses

For primary and recurrent sarcomas, we compared categorical variables using Chi2 test or, if not applicable, Fisher's exact test. Continuous variables were analysed by twoway t-test. Time at risk was calculated as days from the date of surgery to end of follow-up. We constructed Kaplan-Meier survival plots of 5 year mortality and used a log rank test to test for differences between curves. We performed univariate Cox' regression analyses to find predictors for death, using time since date of surgery as the time scale. Variables identified in the univariate analysis as predictors of mortality (using p-value ≤ 0.1 as cutoff) were entered into a multivariate Cox regression model. P-values ≤ 0,05 were considered statistically significant. All analyses were done using STATA 9.2 (Statacorp., College Station, Texas, USA).

## Results

### Short-term outcome

Primary tumors required less extensive surgery than recurrent tumors and could be removed without resection of adjacent organs in 34 of 65 patients (52%) compared to 6 of 22 (27%) (p = 0.04). Correspondingly, resection of two or more organs was necessary in 10 (45%) patients with recurrent tumor and only 7 (11%) with primary tumors (p < 0.001).

In spite of the more extensive and complex surgery, the rate of postoperative complications in the group with recurrent sarcoma was very low, and fully comparable to that of primary sarcoma (Table 2).

**Table 2:** Postoperative complications

	Primary Tumor (n = 65)	1st Recurrence (n = 22)
Anastomosis		
Number of anastomosis	25	10
Reoperation due to leakage	1 (4%)	0
Other intraabdminal complications		
Bleeding	3 (5%)	3 (14%)
Abscess	0	0
Other	3 (5%)	0
Wound complications		
Infection	4 (6%)	0
Wound dehiscense	3 (5%)	1 (5%)
Cardiopulmonary complications	5 (8%)	3 (14%)
Deep venous thrombosis	2 (3%)	0
Death within 30 days	2 (3%)	0

Thirty-day mortality in the recurrent sarcoma group was zero. In the group with primary sarcoma, 2 patients died within 30 days of surgery. For the two groups combined, the 30 day mortality was 2.3% (CI: 0.3-8.1%)

### Long-term outcome

**Figure 1:**
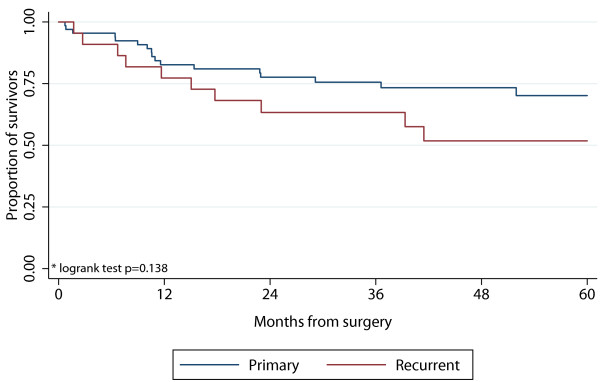
**5-year survival after surgery for intraabdominal or retroperitoneal sarcoma comparing primary and first recurrence sarcomas**.

The 5-year survival rate for patients with a primary tumor was 70.2% (CI:0.56-0.81) compared to 51.8% (CI:0.29-0.71) in patients with recurrent disease (p = 0.138) (Figure 1).

**Figure 2:**
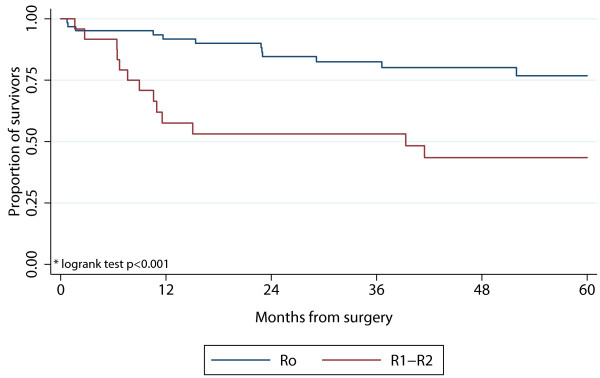
**5-year survival after surgery for intraabdominal or retroperitoneal sarcoma comparing radical (R0) and non-radical (R1 + R2) surgery**.

The 5 year survival rate for patients with R0-excision was 76.8% (95% CI: 0.62-0.86) compared to 43.5% (95% CI: 0.23-0.62) in patients with R1 or R2 excision (p < 0.001) (Figure 2). We found no difference in 5 year survival rates between patients with GIST (63.4%, 95% CI: 0.44-0.77) and non-GIST tumors (56.9%, 95% CI: 0.42-0.69, p = 0.29)).

**Figure 3:**
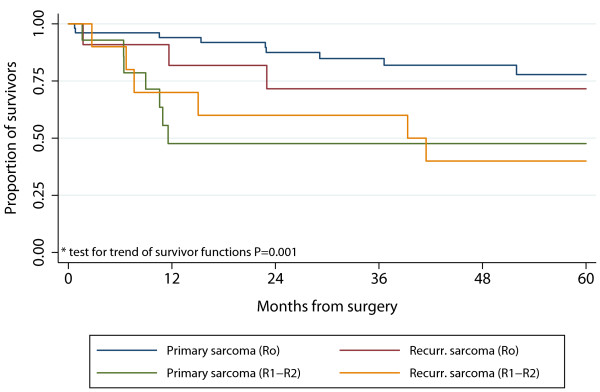
**5-year survival after surgery for intraabdominal or retroperitoneal sarcoma comparing primary and first recurrence sarcomas undergoing radical (R0) and non-radical (R1 + R2) surgery**. Radicality, but not whether the sarcoma is primary or recurrent, is essential for survival.

The Kaplan-Meier plot of primary/recurrent and radical/non-radical surgery is shown in Figure 3. The survival rates of patients having undergone R0 surgery were similar for primary (77.8% CI: 0.61-0.88) versus recurrent sarcoma (71.6% CI 0:.35-0.90). Accordingly, in the multivariate model only age 70+ HR 4.49 (95% CI: 1.78-11.3) and radicality HR 4.39 (95% CI: 1.80-10.7) remained significant predictors of death. Recurrent disease was not an independent predictor of death, no was location or histopathology (GIST/non-GIST).

## Discussion

In patients with IaRS that generally require extensive surgery the best results are expected to be achieved by a multidisciplinary team involving surgeons, radiologists, onchologists, and pathologists in an experienced treatment center [11-14].

In addition to studying 65 patients undergoing surgery for primary IaRS, the study includes 22 contacts with patients with recurrent disease after surgery for IaRS, providing a unique opportunity to explore the outcome in these patients. Few publications looking specifically at this category of patients have been published [4].

In the publications on surgical treatment of IaRS that report these data, the perioperative mortality in primary IaRS is between 3 and 7% [7,15-18]. Non-fatal perioperative complications are reported in 8-44% [15,18,19]. The mortality in our center was comparably low, only two patients (2.3%) died within 30 days of surgery and serious complications were also very rare.

In primary sarcomas, resection of adjacent organs was necessary in 48%, which is in the same order of magnitude as in other reports [7], and radical surgery was achieved in 78%, also comparable to other centers [7]. As expected, in patients with recurrent disease after first surgery for IaRS the disease was more advanced. More often, these patients had metastatic disease and they were assessed to be non-operable more frequently. Although basic surgical techniques were respected, macro- and microscopic radicality was achieved in patients with more advanced disease less frequently, in 50% of cases. In spite of the more extensive surgery, however, peri- and postoperative complications in patients with recurrent disease were not increased, and the 30-day mortality was zero, stressing the importance and impact of optimal intra- and postoperative management.

While the rate of perioperative mortality and complications varied considerably

in other studies, the 5-year survival was remarkably consistent, about 50-55% [13,15-18]. Our

survival rate for primary IaRS was 70.2%, well in line with others. In earlier publications,

surgery for recurrent IaRS has been considered palliative [16]. We found a 5-year survival in

recurrent IaRS of 51.8%, but when looking specifically at those recurrent tumors where

radical excision was achieved, the 5-year survival rose to 71.6%, similar to the survival

rate of primary sarcomas with radical excision. The fact that the radicality of the surgery is

such an important prognostic factor is in line with the conclusions in other studies [4,16,19,20].

To conclude, even when primary curative surgery fails, secondary surgery for recurrent IaRS results in a 51.8% 5-year survival, increasing to 71.6% if a radical resection can be achieved. As such, recurrent disease has the same prognosis as primary if radical surgery is achieved, indeed radicality but not primary/recurrent disease is an independent predictor of death. However, secondary surgery for recurrent sarcoma is often more extensive involving resection of adjacent organs. For such a treatment to be carried through, it is crucial to keep the frequency of peri- and post-operative complications as low as possible, and we report that this can be achieved in a highly specialized surgical center.

## Competing interests

The authors declare that they have no competing interests.

## Authors' contributions

ASS, JL, and SL contributed substantially in all parts of the study except from the collection of data. MS, PW, and PR contributed substantially in the planning of the study and the collection of data as well as in the interpretation of the data. OSS contributed substantially in the analysis and interpretation of the data. All authors reviewed the manuscript and approved the final version.
